# Methods for working with problem residents in medical physics residency education

**DOI:** 10.1002/acm2.70068

**Published:** 2025-03-13

**Authors:** Christopher J. Watchman, Dandan Zheng

**Affiliations:** ^1^ Department of Radiation Oncology University of Arizona Tucson Arizona USA; ^2^ Radiation Oncology Banner University Medical Center Tucson Arizona USA; ^3^ Department of Radiation Oncology University of Rochester Rochester New York USA

**Keywords:** Education, Problem, Resident, Training

## Abstract

Medical physics residency training programs may occasionally encounter residents requiring additional intervention beyond normal training efforts. In the literature, these residents are referred to as “problem” residents. While the physician literature on the subject is valuable, this paper specifically focuses on dealing with a problem medical physics resident. This work discusses a generalized strategy for addressing and correcting medical physics problem resident issues. A discussion of categories of problems that may be encountered is also presented. Additionally, a standardized process for resident intervention is given, along with a discussion of issues related to transparency and bias. Applying the principles in this work should assist medical physics residency programs in establishing a strong culture where all residents, including those experiencing difficulties, can successfully complete their medical physics residency training. This work is a result of collaborations facilitated by the Society of Directors of Academic Medical Physics Programs (SDAMPP).

## 1 INTRODUCTION

Residency education is an exciting and demanding time in the career of a clinical medical physicist. Most residents find the process challenging but progress well through their training. Occasionally, difficulties may arise in training a resident that requires intervention from the program director (PD) to resolve the issues related to the resident. Residents who need this additional intervention are commonly referred to as “problem residents.” A “Problem Resident” is defined as a trainee who demonstrates a significant enough problem that requires intervention by someone of authority, usually the PD or chief resident.^1^ This definition has widely been discussed in the literature for physician training.^2^, ^3^, ^4^, ^5^, ^6^, ^7^, ^8^, ^9^

One of the main challenges that problem residents may pose is ensuring that patient care is not compromised. Medical physics residents play an essential role in delivering radiation therapy, imaging services, and radiation safety programs. Therefore, it is critical to identify and address any performance or behavior issues that could significantly impact patient safety. Problem residents may exhibit poor communication skills, lack of attention to detail, or resistance to feedback, which may increase the risk of errors or delays in patient care. The residency program, with its dedicated professionals, is responsible for identifying and managing these issues to ensure patient care is not compromised.

Another challenge posed by problem residents is their impact on the learning environment. A single problem resident can affect the morale and productivity of the entire residency cohort, creating a negative atmosphere that can impede learning and professional growth. Problem residents can also create conflicts and tension with other healthcare professionals, such as radiation oncologists, radiologists, and nurses, which may compromise collaboration and patient outcomes.

Despite the earlier definition being widely used and the challenges presented by the problem resident, redefining a problem resident as “a resident with a problem” may be more effective in addressing the resident's and the program's needs. While medical residency training shares many similarities with medical physics residency training, there are some significant differences. This work will discuss types of problem residents, a generalized strategy to address a problem resident, categories of resident problems, and specific strategies and tactics in dealing with each category. Other topics relevant to an effective and just adjudication of resident problems will be discussed.

## 2 GENERALIZED STRATEGY

**FIGURE 1:**
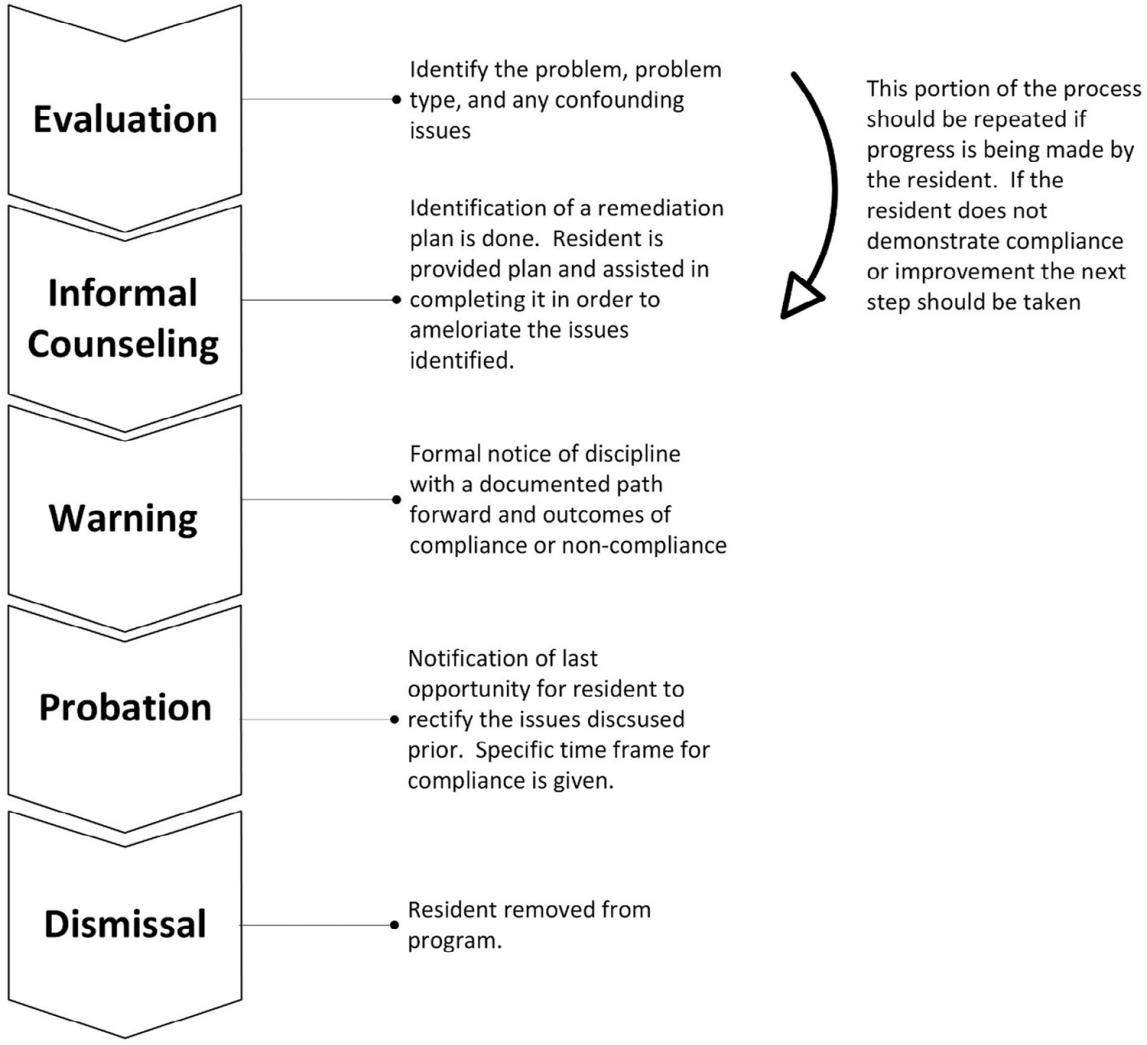
Overview of the generalized resident intervention protocol.

It would be desirable to preemptively screen for problem resident characteristics prior to acceptance to the residency program. In the paper by Taira, Santen and Roberts^9^ a front end approach to problem residents is discussed. Despite proactive attempts, addressing issues with a problem resident may become necessary during residency training. Figure 1 presents an overview of the complete intervention process. Details of each step of the process will be described throughout the paper. The first step in the process is to identify the issue. General categories of these issues will be discussed later in Section II. Identifying the problem may be done by the PD, faculty, or staff. Depending on the program's size and assigned duties, the chief resident may or may not be involved.^4^ If they participate in this step, their efforts should be coordinated under the direction of the PD. Depending on the nature of the problem, the PD may need to include the steering committee, departmental leadership, and human resources. The next step involves a discussion of the problem with the resident. This discussion should be forthright. Efforts should be made to help the resident know that the overall objective of this discussion is to help them progress toward successful graduation and competency as a clinical medical physicist. The goal is to help them improve their performance and prevent negative consequences. The PD/steering committee should consider the resident's point of view regarding the issue before the next step in the process, which is determining corrective action. All salient factors, including mitigating factors, program requirements, resident needs, and patient safety, should be considered during this step. Once the corrective action has been identified, another discussion with the resident should occur where milestones/requirements are clarified to facilitate continued resident progress. These requirements should be clear, achievable, and include a timeline for completion. Poorly delineated corrective actions and deadlines can exacerbate a resident's problem, making it more challenging to resolve the situation positively. Once benchmarks for improvement are set, the PD must evaluate the resident's progress. Several outcomes can come from this evaluation, including but not limited to further intervention, resolution of the problem, and disciplinary actions. The resolution of the problem is the goal of this process. Depending on the issue, going through this generalized process more than once may be required for the resident to resolve the problem satisfactorily. Avoiding serious disciplinary actions needs effort both on the part of the resident and the program, but it may be unavoidable, as will be discussed later.

When this generalized process is used, PDs should adapt it to their needs. Some resident problems may not require corrective action from the resident but from other program members, possibly including faculty, staff, and the PD. In these cases, conflicts of interest may arise. Should this happen, the program steering committee must become involved to prevent conflicts of interest from negatively affecting the resident and the program.

## 3 CATEGORIES OF RESIDENT PROBLEMS

**FIGURE 2:**
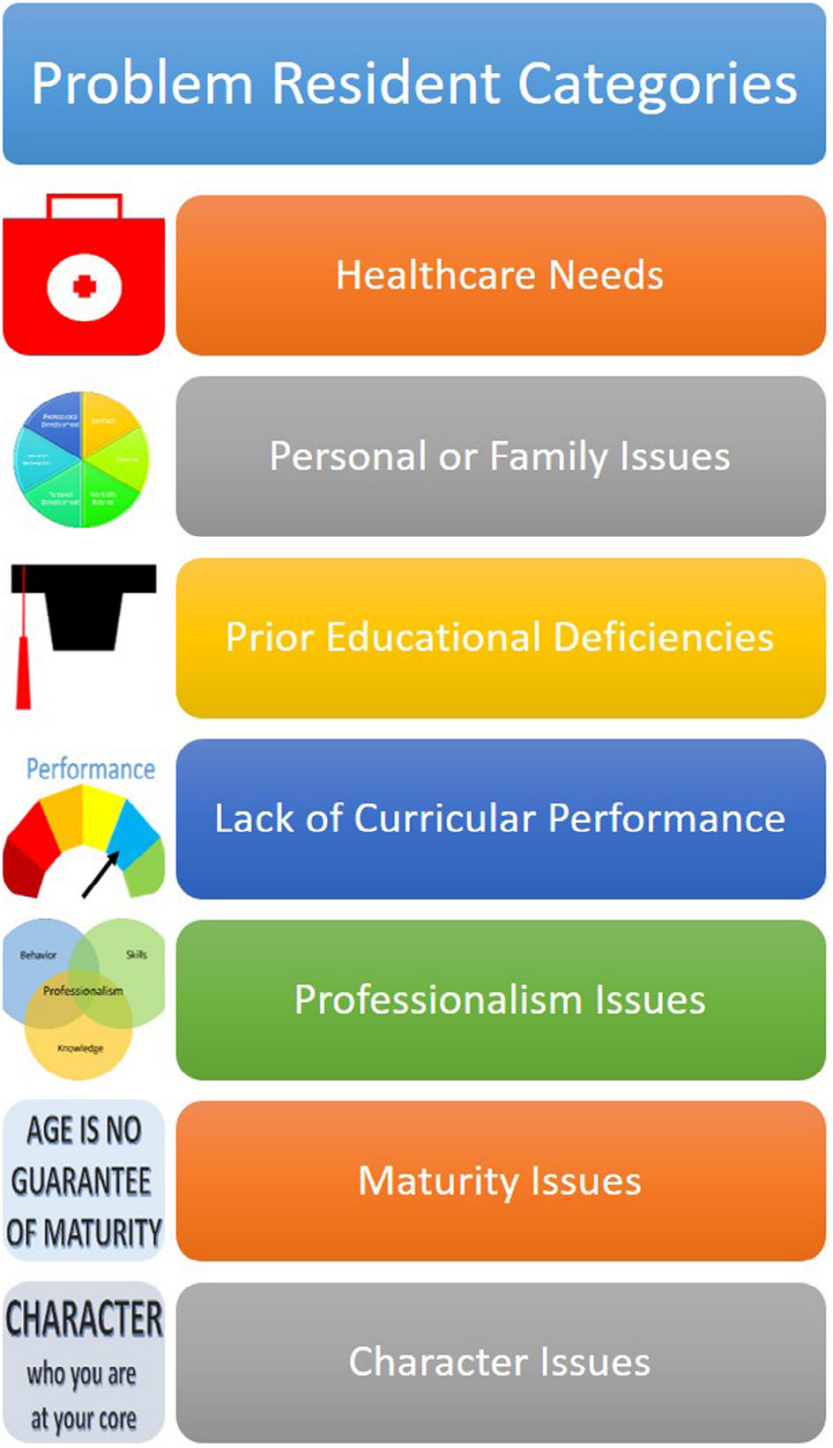
Problem resident categories.

Most problem resident issues can be categorized into seven different types as shown in Figure 2. Some residents may have unique problems or problems that overlap category types. Consequently, each situation should be evaluated individually. The first category is healthcare needs, which may include the resident or the resident's family medical situation. Medical issues may be acute or chronic and require short or long‐term interventions. The second type comprises personal or family matters not related to health concerns. Problems in this category include issues that affect the individual well‐being of the resident. Work‐life balance issues are included in this category. Prior educational deficiencies are the next class of resident problems. Gaps in the resident's education before starting residency may prevent the resident from being fully prepared to understand and internalize the residency curriculum. Gaps in the resident's prior training may be due to the resident's efforts in graduate school, variation in different graduate programs’ curricular emphasis, or other undefined reasons. Issues in this group may lead to the next type of resident problem, inadequate residency curricular performance. Prior educational deficiencies are only one of the possible reasons for a resident's poor performance. Identifying the key reason for the difficulties is critical to determining what corrective action is needed. The next category is professionalism. Professionalism is a combination of knowledge, behavior, and skills. Problems with professionalism will require evaluating the resident in all areas, including but not limited to their communication skills and interactions with fellow residents, staff, and faculty. Maturity is the next class of resident problems. In residency programs, we may have a large range in resident age and experience that can contribute to a lack of maturity. That being said, age is no guarantee of maturity. The last type of resident problem is that of character. Personal character is fundamentally who a person is, meaning it is deeply ingrained in the individual. We can see that each individual is different by looking at the core pillars of character– trustworthiness, respect, responsibility, fairness, caring, and citizenship since each resident will express these traits differently.

Identifying the type of resident problem that must be addressed is vital to a successful resolution. As mentioned earlier, many of these categories may overlap, requiring the PD to assess each situation individually.

## 4 INTERVENTION AND REMEDIATION STRATEGIES

### 4.1 Healthcare

Healthcare needs are a common issue that may require intervention. Residents are not immune from the effects of medical needs during their residency. Consequently, PDs may need to assist the resident in ensuring their successful completion of the training program. Identifying the type of problem is critical when dealing with resident healthcare needs. Healthcare issues may be chronic or acute and/or individual to the resident or due to family needs. Interventions may be short‐term or could last the entire length of the resident's program. When dealing with these issues, we must remember that confidentiality is paramount. We also need to focus on whether the issue will need an ongoing solution or if it may be resolved in a distinct period of time. If the solution is short‐term, one must focus on how the department supports the resident's needs. Longer‐term needs may require additional institutional benefits and support. Helping identify what is available to the resident can provide much‐needed support. Institutional financial support should also be presented to the resident when possible. This could include continued financial support should their residency need to be extended. Additionally, residents should have access to short‐term disability financial support and other standard job protection programs such as the Family Medical Leave Act (FMLA). When the resident's needs are related to family medical issues, the program should try to accommodate the resident's family obligations while balancing the clinical needs of the program.

### 4.2 Personal or work life problems

A resident may also have other issues not related to health that are affecting their well‐being. PDs, faculty, and other staff members associated with the residents must always look at the resident as a whole person who needs to balance their life. What “balance” means may differ for each resident, so each resident should be treated individually while balancing the program's needs. Papers from Di Tella et al.^10^ discussing quality of life issues with respect to empathy., Paradis et al.(2021)^11^ covering work life integration, and Paradis et al. (2022)^12^ expounding on resilience and well being, may help understand resident problems in this area. An example of these types of issues may include financial concerns. The program may provide financial education opportunities to help improve the situation if appropriate. Other options might be to allow residents to moonlight or do additional paid work beyond their residency duties. For example, treatment planning overflow may be mitigated by a pay‐per‐plan option. We must also recognize that some family obligations regarding children or aging parents may require schedule adjustments that allow the resident to fulfill their family and residency obligations.

Another area of concern is burnout. This is a current topic in medicine as many healthcare workers, including medical physics residents, suffer from burnout.^13^, ^14^ PDs must be aware of the potential for resident burnout, which can lead to negative results, including but not limited, to loss of empathy, and medical errors.^10^, ^14^ PDs should regularly check in with their residents, and if burnout is identified, the PD may suggest the use of vacation time and/or a change in the residents’ duties and learning schedule. Another option would be to provide a “compensation day” if the resident's work hours become excessive and lead to burnout. It should be noted that with all of the suggestions above, program resources may not allow for some of these solutions and may require solutions unique to the program. Additionally, when addressing the problem resident's needs, program fairness should be considered. Equitable application of solutions, such as compensation days, should be applied to all residents in the program.

### 4.3 Prior education deficiencies

Despite adopting the Commission on the Accreditation of Medical Physics Education Programs (CAMPEP) curriculum standards at the graduate and certificate level (campep.org/GraduateStandards.pdf), a resident may enter a residency program with gaps in knowledge and understanding. Deficiencies may result from differences in program implementation of the CAMPEP curriculum, differences in faculty focus, differences in how the student learns compared to the instructor, and individual learning approaches on the part of the student.

CAMPEP curriculum standards (campep.org/GraduateStandards.pdf) and those provided by the American Association of Physicists in Medicine (AAPM) task groups provide an extensive list of topics that should be covered by the graduate or certificate programs.^15^ The topics listed in the curriculum guide should provide an incoming resident with a solid didactic background for residency training. Using these guidelines, each program develops its curriculum and, in doing so, creates a unique educational environment. Different programs will have faculty with different strengths and interests. This variability may result in varying degrees of depth and focus on each topic listed in the curricular requirements for accreditation. This is not to say that the curriculum implementation by the graduate or certificate program or it's instructor is deficient, but instead that these differences may result in the resident's incomplete understanding of a particular topic.

Another area where a barrier may occur is differences in how the instructor learns compared to the student's learning style. A cognitive disconnect may result from these differences as the student can have greater difficulty assimilating the information. It can be challenging for instructors to adapt to differences in learning styles, but they should try to accommodate different learning styles into their teaching. An additional factor may also result in learner deficits due to changing generational learning norms. It has been shown that Millennials and Generation Z students have different learning approaches than previous generations.^10^, ^13^ This disconnect can result in learning deficiencies that can cause a resident difficulty during their training.

While curriculum implementation and learning style differences may play a role, often, it is the student who was unsuccessful in assimilating the information. When this happens, it is generally due to the following reasons, according to Gibbs (1979), “(1) students lack the necessary study skills; (2) students are of different types, and some student types have limited learning approaches; (3) students choose their approaches to studying, some of which are ineffective or inappropriate; (4) students develop in their sophistication as learners, and some are less developed than others, and (5) students are held back in their learning”.^16^

Due to the reasons discussed, the residency PD and associated faculty are responsible for identifying these deficiencies as early as possible during the resident's training. This can be a complex process that will often require in‐depth discussions with the resident beyond the normal evaluation process (exams, practicals, etc.) to identify the root of the problem. In some cases, the problem may be easily identified and corrected. A more definitive and structured remedial training plan must be developed in others. Early detection of the deficiency is more likely to result in a positive outcome for the resident. Consequently, continual formal and informal evaluation of the resident should be undertaken.

### 4.4 Lack of residency training performance

Closely tied to the pre‐residency curricular deficiencies problem resident is the resident who fails to perform up to the program curriculum standard. Prior curricular deficiencies may contribute to a resident's failure to perform, but they may be due to other reasons related to their understanding of residency topics. The key to identifying and correcting these problems is similar and should focus on regularly evaluating the resident's performance. Once identified, specific remediation training and key performance milestones should be presented to the resident.

### 4.5 Professionalism

Problems with professionalism may also arise, and the PD may need to address them. Professionalism is a combination of knowledge, behavior, and skills that will require evaluating the resident in all areas of professionalism, including but not limited to their communication skills and interactions with fellow residents, staff, and faculty. The key to addressing professional issues is having a frank and open discussion with the resident once the primary issue is identified. During this conversation, the PD should express concern for the resident and provide corrective actions the resident should take to improve. The PD may need to provide the resident with additional resources to train the resident. A designated professionalism mentor, either the PD or another faculty member, may need to be assigned to help the resident develop their professional skill set. Some institutions may be able to provide additional training courses in professionalism, communication and so forth. Additional resources provided by the AAPM, include a multi‐institutional journal club on professionalism and ethics,^17^ the Medical Physics Leadership Academy,^18^ and the AAPM/RSNA Professionalism modules.^19^ Setting milestones will also help the resident by giving them specific goals or actions to strive for. These milestones should be evaluated; if the resident shows improvement, further intervention is not needed. For some residents, the next step may be repeating the process until rectified, or disciplinary actions may need to be taken.

### 4.6 Maturity

A resident's level of maturity or immaturity is another category that may require PD intervention. In residency programs, we may have a broad range of resident ages that can contribute to a lack of maturity. That being said, age is no guarantee of maturity. We must remember that maturity is a product of life experience. When addressing issues in this area, the PD should remember that generational differences may affect maturity expectations.^16^, ^20^, ^21^ Care should also be taken to evaluate the resident's life experience. Keeping this in mind, the intervention may only require a simple discussion on expectations for the resident. Other interventions may require addressing specific situational examples to help build their experience.

### 4.7 Character

The next key category of problem residents is character. Personal character is fundamentally who a person is, meaning it is deeply ingrained in the individual. We can see that each individual is different by looking at the core pillars of character– trustworthiness, respect, responsibility, fairness, caring, and citizenship. Each resident will express these with differences in their individual traits. Interventions by PD to address character issues may be very difficult. The literature shows that interventions addressing character issues have a low probability of success.^1^, ^2^, ^3^, ^4^, ^5^, ^6^, ^7^, ^8^, ^9^ Despite that, when successful, they often impact the resident's life the most. One of the reasons why dealing with these issues is problematic is that character development takes real effort by the person making the change. The PD should help the resident identify their weak areas and provide guidance on improving. The PD may also need to direct the resident to other resources, whether within the institution or outside, to raise the potential for success.

## 5 PROBLEMS SPECIFIC TO MEDICAL PHYSICS TRAINING

Medical physics residency training has distinct differences from medical residency training programs. These differences include shorter time durations, technical and clinical training, and variation in training programs. Medical physics residencies are much shorter, approximately half the time, than radiology or radiation oncology residencies. This shorter time frame presents significant challenges in addressing problem resident issues. Early identification of a resident's problem and specific issues is necessary; otherwise, timely intervention may not be possible.

When issues are not identified until the later stages of a program, remediation efforts may introduce a temporal stressor to the resident that could further complicate correcting the problem. The PD should develop a mechanism where they may be able to identify a problem resident early in the resident's training program.  Feedback from training mentors is essential in determining this. In some circumstances, training mentors may be reluctant to address their concerns with the PD regarding resident deficiencies. Faculty/staff may be concerned about doing more harm than good for the resident by sharing their worries. Additionally, faculty or staff may have fears regarding retaliation. PDs need to assuage these concerns and can do so by creating an open and honest feedback mechanism that maintains the privacy of both the training mentors and the residents. The PD should also reinforce to mentors that early intervention almost always has a better outcome for the resident than later interventions, especially considering the limited timeframe of medical physics residencies.

Additionally, the shortened residency timeframe often results in residents developing work‐life balance problems where they over‐prioritize their training as they know they only have a short time to learn.  Consequently, vacations are frequently neglected, resulting in additional stress on the resident, which may affect their long‐term and short‐term mental health.  The PD should address these work‐life balance issues early with simple discussions noting the necessity of time off and recovery. Another matter related to the short time frame of medical physics residencies is that any deviation from the training schedule due to intervention and remediation efforts will likely require an extension of the resident training time to complete all training requirements. This could provide additional financial and mental stress on the resident, further impacting their performance.  PDs should ensure that all training requirements are completed while mitigating financial stress.

Another issue that can result in a problem resident is insufficient technical preparation before starting residency. Weak technical preparation and clinical applications can often be identified early.  Despite this, the shorter duration of the medical physics residency may not allow for significant remediation intervention without delay of the program completion.  Medical physics residents are often tasked with clinical duties after hours. This results in medical physics residents working a standard work day during clinic hours and additional hours after clinic closure. Out‐of‐clinic activities may result in reduced study time for medical physics residents. When addressing deficiencies in preparation, the PD should develop a resident improvement plan that considers these realities.

## 6 STANDARDIZED PROCESS IN RESIDENT INTERVENTIONS

In light of the above discussions, a program should have a clearly defined process for handling problem residents and clear delineations of situations that may trigger the due process. These policies should be fully communicated to the resident during the onboarding or even the application process. Developing a well‐thought‐out policy and due process before having to deal with its first problem resident often helps a program and the resident get the best outcome when such situations arise. Having a clear mutual understanding of expectations at the beginning also helps avoid and alleviate potential problems.

The process must be delineated to effectively deal with situations where continuous underperformance or professionalism/character issues are identified. Disciplinary actions should be conducted following a defined policy. The process may vary among different programs, depending on their institutional policies, whether the program is governed by the institution's Graduate Medical Education (GME) office or the policies are designed by the individual program. Problem‐resident policies usually contain some or all of the following disciplinary actions.

1. *Evaluation*. A written (including electronic) evaluation of the resident addressing knowledge, clinical competence, performance, professionalism, interpersonal and communication skills, and ethical behavior. Specific resident deficiencies and improvements should be delineated.
2. *Informal counseling*. This step is usually considered not reportable and is not considered a formal disciplinary action. Actions may include a verbal or written warning. These actions may be divided into sub‐steps: minor informal counseling (verbal and no records kept) or moderate informal counseling. A memo will be placed in a file outside the resident's record for reference and tracking by the PD.
3. *Warning*. An official notification requires the delivery of an administrative status letter or an official warning letter. This letter and any documents related to its resolution are kept in the resident's file for the duration of the residency. This action should be seen as a strong indication that the resident is on unsteady ground and that the next step is formal disciplinary action. This letter should include an expectation of improvement for the specific issue (conduct or performance). The required corrective action should be delineated along with a specific timeline for the correction to be completed. This letter should be documented in the resident's file, and copies sent to other appropriate groups in the institution, that is, human resources or GME.
4. *Probation*. This step is usually the last opportunity for the resident to correct themselves before dismissal. Note that the first step of formal disciplinary action is reportable to credentialing and licensing agencies. A formal record of the violation must be maintained, along with a remediation plan that includes evaluation criteria and a timeframe. Failure to meet the goals and requirements within the prescribed timeframe will result in dismissal from the residency program. This record is maintained permanently in the resident's file and with any other required institutional department. The length of probation may vary from program to program, but it should be thoughtfully designed to fit the residency training length and give the resident reasonable time to make corrections. During this period, intensified remediation and evaluation are often involved. It is essential to document remediation actions taken by both the program and the resident during this formal disciplinary action and the following assessments. At the end of the probationary period, if the resident has completed the probation requirements, they may be taken off of probation and continue in the program. If unsuccessful, extended remediation, additional probation, non‐renewal, denial of the certificate, or termination may be necessary.
5. *Dismissal*. Dismissal could occur if the resident fails to achieve the requirements delineated in the preceding disciplinary action step. Programs may use different discharge forms such as voluntary resignation, involuntary termination, non‐renewal of appointment, and denial of residency completion certificate. Should the resident wish to seek grievance if dismissed or their contract is not renewed, the program should also delineate a formal grievance procedure. The program may also dismiss a resident without probation in rare instances of flagrant or malicious misconduct. Immediate dismissal would be for conduct beyond that which is not considered professionally acceptable and in any way denigrates or endangers an individual, the reputation of the department/institution, or willfully places a patient in harm's way.

The process should be established with well‐defined evaluation criteria and procedures and communicated to residents at the beginning of their training. Individual programs may have varying acceptable learning metrics and clinical competence measures, but detail and structure are generally helpful, and so is an upfront definition of evaluation mechanisms and timeframes. Although efforts are consistently made throughout the residency program to aid the resident in progressing toward completion, the above disciplinary actions may be necessary to ensure the competence and professionalism of graduates from the program. It is always preferred to have a well‐defined process and procedure in dealing with such situations. The process should be transparent to both the resident and the program staff. In this manner, remediation could be implemented most efficiently, and conflict or dispute could be avoided or minimized.

Some of the actions in this process, such as extended remediation, may lead to extending the duration of program completion. This is particularly the case for medical physics residency programs because of the relatively short duration of the 24‐month clinical training. There may be practical considerations that need to be addressed. Some examples include the maximum allowed months of the lengthened program; the extension of salary, benefits, and clinical access; The American Board of Radiology (ABR) exam eligibility due to delayed completion; residency training capacity due to the out‐of‐cycle extended training; and other institutional training program compliance issues that arise from the situation. Preemptive planning may be helpful in these cases.

Generally, the final disciplinary step, dismissal, should only be taken against a resident with the approval of the Residency Steering Committee, the PD, and the department leadership. This consensus is not superficial but is intended to assure that the facts and circumstances fully warrant dismissal. Including the steering committee ensures that the resident involved has been given every reasonable consideration before this decision is made. If a resident believes that a condition of their training or disciplinary action is unjust, inequitable, or a hindrance to effective operations or performance, they may initiate a grievance. It is advised that the program specifies the people involved in the grievance procedure and the formal process of resolving the grievance.

## 7 CULTURAL AWARENESS AND UNCONSCIOUS BIAS

Objective evaluation of resident performance is an integral part of the discussion addressed in this article. Both clinical and professional competence must identify the meaning of competence and how it is measured. Cultural awareness and competence should be considered in this process, and efforts should be made to develop a multicultural mindset in the program.^22^, ^23^ This is important for workplace diversity and inclusion and for successfully training residents with cultural competence in the health care service they provide.

The first step towards cultural competence is cultural awareness through exploring unconscious or implicit bias. Unconscious bias is often incompatible with one's conscious value because one is unaware of having such bias. However, unconscious bias can be far more prevalent than conscious prejudice. An example of unconscious bias in the health care setting given by Howard Ross, a famous writer on bias, is about the higher dehydration rate and lengthened postpartum hospitalization for Vietnamese women in American hospitals. Ross discussed how that the dehydration and subsequent adverse effects stemmed from the Vietnamese cultural tradition of avoiding cold drinks in situations that involve hemorrhaging, especially childbirth. Therefore, postpartum Vietnamese women usually turn down cold beverages offered in American hospitals during and after delivery, leading to dehydration.^24^ Understanding the unconscious factors could help resolve the significant issue by offering drinks in the preferred temperatures.

Similarly, unconscious bias in the healthcare workforce and residency training may exist if we only assume a single‐cultural mindset and fail to explore the differences in race, gender, culture, personality, and experience.^25^, ^26^, ^27^ For example, oral exams are often an important evaluation mechanism for residents' knowledge and clinical competence. How a resident approaches and paces through an oral exam question, their confidence level and the perceived confidence level, body language, and their interactions (or lack of) with the examiner(s) during the exam may all be influenced by the abovementioned factors. As another example, resident interactions and communications with patients and other clinical staff are critical to professionalism—soft skills that are often informally taught and evaluated in residency. Still, they can also be heavily influenced by those differences. Exploring possible unconscious bias and better aligning the resident's and program's understanding and expectations is important regarding resident underperformance.^28^ Raising the awareness of our personal unconscious biases will go a long way to reducing their effects. Open communication and early intervention are especially critical for the short training duration. Medical physics in the United States has historically been composed of talents from diverse cultures and backgrounds. Therefore, improving cultural awareness and competence and addressing unconscious bias in residency training should not be a burden or hindrance but an opportunity to capitalize on the diversity we enjoy in our profession and an opportunity to serve patients better.

## 8 PROGRAM CONTINUITY AND FITNESS

Successful residency programs may have different strengths, but usually, one factor is common: mutual respect. The respectful relationship within a department and a team are essential, resulting in integrity, understanding, cooperation, and team spirit. It also uplifts a residency program as the residents feel the faculty has their best interests in mind when dealing with them. If a resident's ethical or behavioral problems go uncorrected or any competence deficiencies are unaddressed, it could lead to distrust among the clinical team members. Uncorrected problems can worsen existing issues for the resident, crush the spirit of fellow residents, diminish accountability in clinical culture, and ultimately be a disservice to and possibly harm the patients.

Many programs rely on a strict budget to support the defined length of resident training. However, the flexibility to extend the training is helpful and even necessary when a resident fails initially but can make up in the long run or when a resident takes extended parental or medical leave. The 24‐month clinical training may be too short to adjust and compensate for such lengthy interruptions. Therefore, it is advised that the program plan for such situations and clearly define the financial responsibilities so that the best quality training is provided and ensured.

## 9 CONCLUSIONS

Medical physicists are not only technology innovators but also the quality safeguards of our medical specialties. Guaranteeing our graduating residents' professional integrity and clinical competency is essential for the health of the medical physics profession and radiation oncology, radiology, and nuclear medicine. For our relatively short 2‐year clinical training duration, clearly defined expectations, evaluation feedback mechanisms, and disciplinary action policies should be given to the residents at the start of their training. This often helps to prevent problems or underperformance in the first place and helps remedy them quickly if they do occur. When the above items are successfully implemented and executed in a residency program, they could also help build stronger teams in which residents learn how it feels to work in a well‐organized clinical practice and get inspired to incorporate the excellent culture and practice into their lifelong careers.

## AUTHOR CONTRIBUTIONS

Christopher J. Watchman, PhD and Dandan Zheng, PhD each contributed to the writing, editing, and completion of this manuscript.

## CONFLICT OF INTEREST STATEMENT

The authors declare no conflicts of interest.
